# Genome organization in cardiomyocytes expressing mutated A-type lamins

**DOI:** 10.3389/fcell.2022.1030950

**Published:** 2022-10-07

**Authors:** Marie Kervella, Maureen Jahier, Albano C. Meli, Antoine Muchir

**Affiliations:** ^1^ PhyMedExp, University of Montpellier, INSERM, CNRS, Montpellier, France; ^2^ Sorbonne Université, INSERM U974, Institute of Myology, Center of Research in Myology, Paris, France

**Keywords:** LMNA, nuclear lamins, cardiomyopathy, genome organization, LADs, lamina-associated domains

## Abstract

Cardiomyopathy is a myocardial disorder, in which the heart muscle is structurally and functionally abnormal, often leading to heart failure. Dilated cardiomyopathy is characterized by a compromised left ventricular function and contributes significantly to the heart failure epidemic, which represents a staggering clinical and public health problem worldwide. Gene mutations have been identified in 35% of patients with dilated cardiomyopathy. Pathogenic variants in *LMNA,* encoding nuclear A-type lamins, are one of the major causative causes of dilated cardiomyopathy (i.e. *CardioLaminopathy*). A-type lamins are type V intermediate filament proteins, which are the main components of the nuclear lamina. The nuclear lamina is connected to the cytoskeleton on one side, and to the chromatin on the other side. Among the models proposed to explain how *CardioLaminopathy* arises, the “chromatin model” posits an effect of mutated A-type lamins on the 3D genome organization and thus on the transcription activity of tissue-specific genes. Chromatin contacts with the nuclear lamina *via* specific genomic regions called lamina-associated domains lamina-associated domains. These LADs play a role in the chromatin organization and gene expression regulation. This review focuses on the identification of LADs and chromatin remodeling in cardiac muscle cells expressing mutated A-type lamins and discusses the methods and relevance of these findings in disease.

## Introduction

**FIGURE 1:**
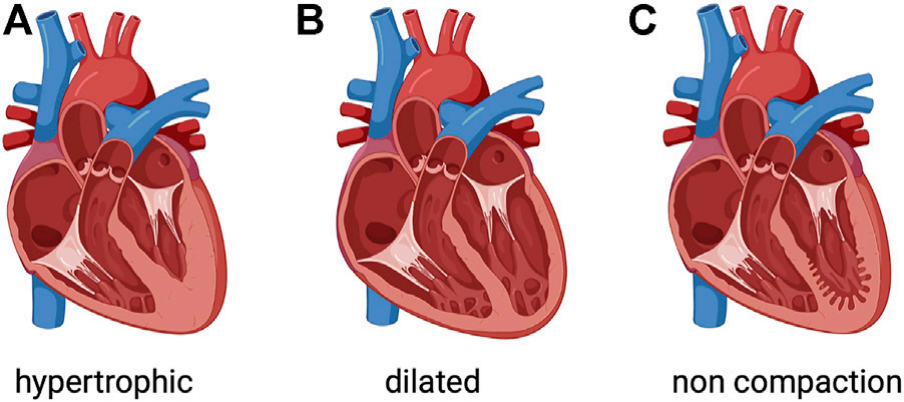
Phenotypic groups of inherited cardiomyopathies. **(A)** Hypertrophic cardiomyopathy involves thickened myocardium, which affects the septum. **(B)** Dilated cardiomyopathy is characterized by a dilated left ventricle with or without right ventricular involvement. **(C)** Left ventricular noncompaction is characterized by a noncompacted endocardial layer surrounded by a compacted epicardial layer with marked trabeculation. Created with BioRender.com.

The global prevalence of heart failure is approximately 26 million patients and the economic load related to this condition is approximately US$120 billion (Groenewegen et al., 2020). The large burden of heart failure implies that developing compelling management strategies is paramount. Cardiomyopathy is a condition associated with contractile dysfunction of the heart, often leading to heart failure. Cardiomyopathies are a clinically heterogeneous group of cardiac muscle disorders, which can be either genetic **(** Figure 1) or systemic (Schultheiss et al., 2019; Hershberger et al., 2021). Dilated cardiomyopathy, the most common form, has an estimated prevalence of >0.4% in the general population. Dilated cardiomyopathy is a condition where the heart muscle becomes enlarged and weakened, resulting in poor left ventricular function defined as a left ventricular ejection fraction <45% (Ziaeian and Fonarow, 2016). Despite current strategies to aggressively manage dilated cardiomyopathy, it remains a common cause of heart failure and a reference for cardiac transplantation. Notwithstanding progresses in reducing heart failure-related mortality, hospitalizations for heart failure remain very frequent and rates of readmissions continue to rise. Presumably, it is important and necessary to increase our knowledge on the pathophysiology of cardiomyopathies to unveil novel molecular/cellular mechanisms for future therapeutic approaches (Leviner et al., 2015).

## Cardiolaminopathy

Among the most common genes implicated in dilated cardiomyopathy, it has been estimated that mutations in *LMNA* gene accounts for about a 10th of familial dilated cardiomyopathy, thus representing one of the major causative genes (Taylor et al., 2003). Affected patients often exhibit early conduction defects before left ventricle dysfunction and dilatation occur. *CardioLaminopathy* usually presents in early to mid-adulthood with symptomatic conduction system disease or arrhythmias, or with dilated cardiomyopathy. *CardioLaminopathy* has an intrusive clinical progression with higher rates of aggressive arrhythmias and faster course towards heart failure than most other cardiomyopathies (Taylor et al., 2003). Given the increased awareness among physicians, cardiologists now use defibrillators in order to avoid sudden death from aggressive ventricular arrhythmias, and pharmacological interventions to improve heart failure symptoms (Meune et al., 2006). Once dilated cardiomyopathy is clinically detected, the management for *CardioLaminopathy* follows the standard of care for heart failure. It is unclear whether early use of these therapeutic drugs prior detectable cardiac dysfunction can modify the aggressive nature of *CardioLaminopathy*. There is no definitive treatment for the progressive cardiac dilatation and loss of contractility in *CardioLaminopathy*, short of heart transplantation.

## Nuclear A-type lamins

**FIGURE 2:**
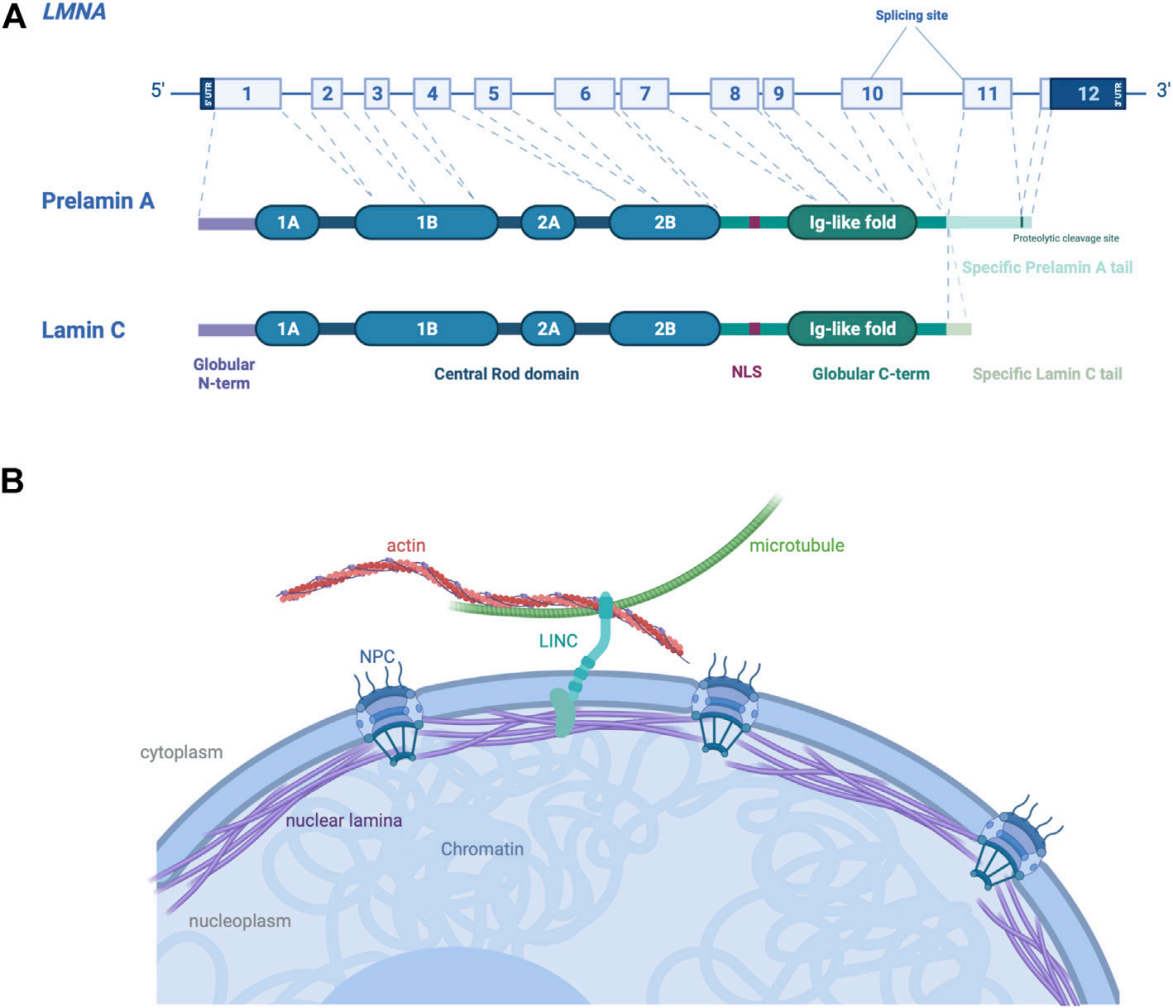
Schematic representation of A-type A/C and their localization in cells. **(A)** *LMNA* gene encodes the A-type lamins, with prelamin A and lamin C generated by alternative RNA splicing being the major somatic cell isoforms. **(B)** Schematic view of A-type lamins, proteins of the nuclear lamina on the inner aspect of the inner nuclear membrane, cause autosomal dominant EDMD. The interactions between A-type lamins with SUN and KASH proteins form the LINC complex, connecting the nucleus to cytoskeleton in the cytoplasm of somatic cells. Created with BioRender.com.

*LMNA* encodes nuclear A-type lamins. Lamins are class V intermediate filament proteins forming the main component of the nuclear lamins, a fibrous meshwork underlining the inner nuclear membrane of most eukaryotic cells (Wong et al., 2022). In mammalian somatic cells, four major lamin isoforms are found, encoded by two different genes. *LMNB1* and *LMNB2* genes encode lamins B1 and B2 respectively, which are ubiquitously expressed in cells during development. *LMNA* gene encodes A-type lamins, lamins A and C, obtained *via* an alternative pre-mRNA splicing **(** Figure 2A), which are mainly expressed in most differentiated cells (Dittmer and Misteli, 2011). A-type lamins interact both with the cytoskeleton in the cytoplasm through the LINC complex (Linker of nucleoskeleton and cytoskeleton), and with the chromatin in the nucleoplasm (Méjat, 2010) (Figure 2B). The LINC complex is a large protein complex composed of SUN and KASH domain proteins, present at the inner and outer nuclear membrane respectively (Haque et al., 2006; McGee et al., 2006). SUN proteins interact with several components of the nucleoskeleton, whereas KASH proteins interact with cytoskeleton through their large cytoplasmic protein domains (Jahed et al., 2021). The LINC complex has been shown to be involved in several biological processes including meiosis, DNA damage repair and gene expression (Wong et al., 2021b). By its location and protein interactions, the LINC complex provides a physical continuum between cytoskeleton and nuclear proteins, allows to withstand and transfer forces across the nucleus (Lombardi et al., 2011).

Given their interaction with LINC complex and chromatin, A-type lamins are involved in a plethora of biophysical and biochemical processes from the extracellular environment to the nuclear interior. A-type lamins participate to the signal mechanotransduction and regulation of the nucleus stiffness and shape (Broers et al., 2004; Lammerding et al., 2004), as well as in chromatin organization, gene regulation, DNA replication, RNA splicing and genome protection from mechanical cues (Dechat et al., 2008; Shimi et al., 2008; Bertero et al., 2019a; Cho et al., 2019).

Cardiomyocytes, by their intrinsic properties, are constantly subject to mechanical stress. Hence, the nuclei must resist these mechanical cues and correctly transmit the mechanical signal inside the nuclei to convert into a biochemical signal and modulate gene expression. In cardiomyocytes, the level of A-type lamins is finely regulated in response to a mechanical stimulus, to prevent nuclear rupture and protect the genome integrity (Cho et al., 2019). Mutations in A-type lamins could alter the signal mechanotransduction and thus contribute to the pathogenesis of *CardioLaminopathy* (Jaalouk and Lammerding, 2009). The mammalian nucleus is a highly specialized organelle, which maintains the genome integrity. The nuclear lamina plays an essential role of mechanotransducer, mechanosensor and participate to the chromatin organization. However, the specific mechanistic roles of A-type lamins in this last process in a cardiomyocyte remain obscure (Carmosino et al., 2014). In this review, we discuss recent findings supporting the role of A-type lamins in the organization and regulation of chromatin in *Cardiolaminopathy*.

## Chromatin compartmentalization in mammalian cells

**FIGURE 3:**
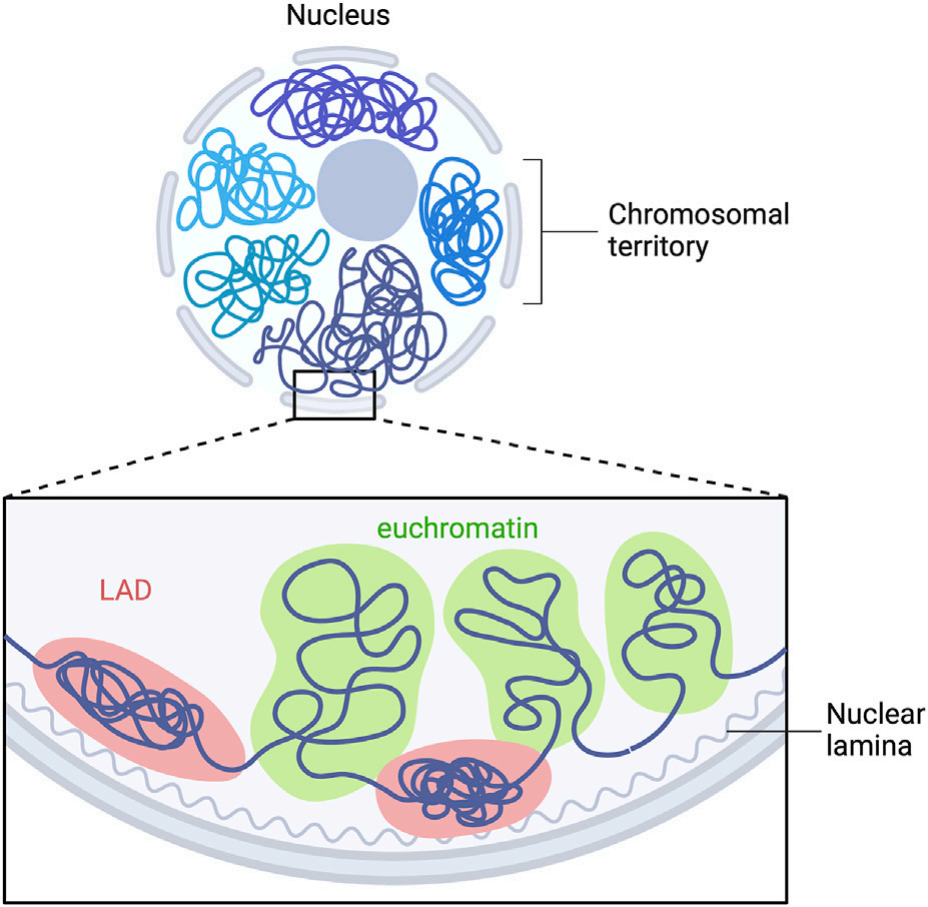
LADs organization in mammalian cells. Model depicting the interaction of multiple LADs with the nuclear lamina. Repressed LADs are located at the nuclear periphery and are composed of “gene-desert” regions, which are enriched in heterochromatin marks. Euchromatin is located within the nucleoplasm and is transcriptional active. Adapted from “Chromosome Organization in Nucleus: TADs”, by BioRender.com. Retrieved from https://app.biorender.com/biorender-templates.

A-type lamins have been shown to be involved in genomic organization (Guelen et al., 2008; Paulsen et al., 2018; Guerreiro and Kind, 2019), recruitment of epigenetic regulators (Auguste et al., 2020) and gene expression (van Steensel and Belmont, 2017). In mammalian nucleus, the genome is highly organized in a tissue-dependent manner. Each chromosome is located in a distinct region called “chromosome territories” (Figure 3) (Cremer and Cremer, 2010), containing two different chromosomal compartments, named A and B (Lieberman-Aiden et al., 2009). A-compartments are gene-rich chromatin regions, enriched in active chromatin marks (H3K36me3, H3K79me2, H3K27ac and H3K4me3) and are preferentially embedded in the center of the nucleus. B-compartments are poor-gene and repressive chromatin marks enriched at the nuclear periphery (H3K9me2/3, H3K27me3) (Bertero and Rosa-Garrido, 2021). The only genes present in B-compartments are silenced or weakly transcribed (Guelen et al., 2008). In B-compartments, large chromatin domains bound to nuclear lamins are referred as *lamina-associated domains* (LADs) (Figure 3) (Guelen et al., 2008; Kind et al., 2013; Amendola and Steensel, 2015; Leemans et al., 2019).

In mammals, A-type lamins interact with hundreds of LADs, which are regions ranging from 0,1–10 megabases in size detected on all chromosomes (Briand and Collas, 2020). LADs were first mapped by DNA adenine methyltransferase (DamID) identification in *Drosophila* (Steensel and Henikoff, 2000; Pickersgill et al., 2006) and later by chromatin immunoprecipitation sequencing (ChIP-seq) (Lund et al., 2014, 2015; Gesson et al., 2016; Cheedipudi et al., 2019; Shah et al., 2021). LADs are predominantly located at the nuclear periphery and are composed of “gene-desert” regions enriched in heterochromatin marks (Kind et al., 2013; Harr et al., 2015).

During mitosis (Wong et al., 2021a) or disease (Cheedipudi et al., 2019), LADs can be reorganized leading to a compartment change and aberrant gene expression (Zheng et al., 2018; Kim et al., 2019) (Figure 2). A-type lamins have been traditionally seen as associated with heterochromatin and transcriptional silencing. However, recent genome-wide approaches show that A-type lamins could be associated with promoters and enhancers outside the LADs, and regulate transcription and chromatin topology of key differentiation gene programs (Leemans et al., 2019).

## Methods to identify and visualize LADs

A comprehensive knowledge of the structural features and dynamics of chromatin is essential to understand cellular mechanisms involving DNA. Though, little is known about the dynamics of chromatin arrangement despite that the scientific community has gained important insights into the higher-order spatial organization of eukaryotic genomes. Several methods have enabled the discovery of higher-order chromatin structure, and notably to study and visualize the LADs organization within the nucleus.

**FIGURE 4:**
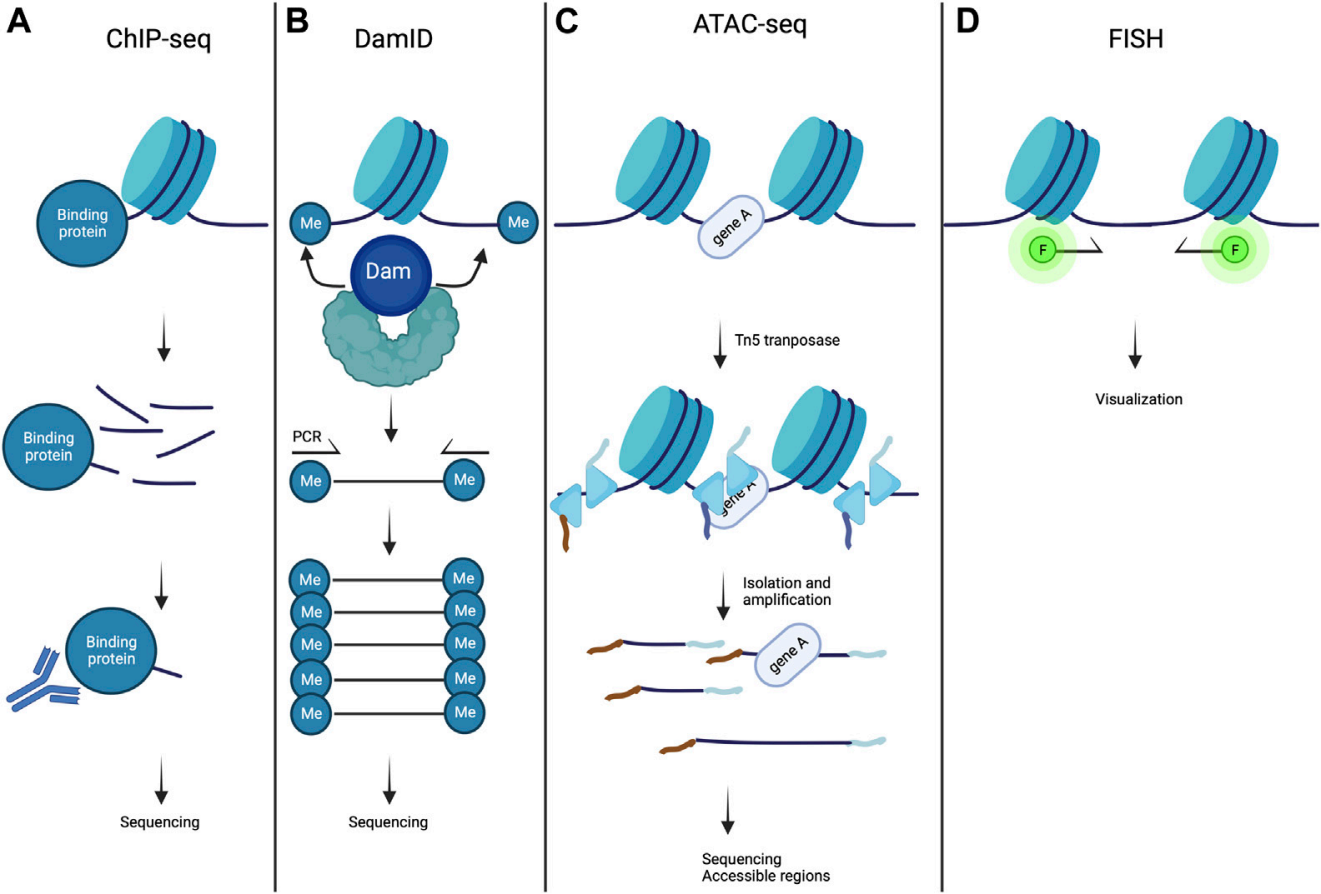
Methods to study chromatin organization. **(A)** In ChIP-seq experiments, chromatin is fragmented and magnetic beads conjugated to antibody specific to the target protein are used to precipitate chromatin fragments bound to the protein of interest. This is then followed by sequencing and mapped onto the genome assembly. **(B)** In DamID, a fusion protein is created consisting of dam methyltransferase and a protein of interest, resulting in local methylation of adenines in GATC sequences on the chromatin. The genomic methylation pattern can be mapped using sequencing. **(C)** ATAC- seq is a methodology used for identifying open chromatin regions. Chromatin is incubated with Tn5 transposase to simultaneously fragment and index the exposed DNA fragments. This is then followed by sequencing. **(D)** In FISH methods, DNA loci of interest are labeled with immunofluorescent DNA sequence specific or chromosome paint probes and are visualized by fluorescent microscopy. Created with BioRender.com.

A large variety of proteins bind to specific parts of the genome to regulate gene expression and chromatin structure. In ChIP experiment **(** Figure 4A **)**, the interacting part of DNA with a protein of interest (lamin A/C for example) are chemically cross-linked together, and DNA is sheared into small fragments. Next, an antibody specific to the protein of interest is used to extract the DNA-protein complex by immunoprecipitation and the extracted DNA sequences are identified by sequencing (ChIP-seq) approaches (Park, 2009).

DamID is a powerful method used to map the genomic interaction sites of these proteins (Figure 4B) (Greil et al., 2006). It is based on fusing a protein of interest to *Escherichia coli* DNA adenine methyltransferase (Dam). Expression of this fusion protein *in vivo* leads to the addition of a methylation group to the adenine in GATC sequences (adenine-6-methylation (^m6^A)). Because adenine methylation does not occur endogenously in eukaryotic cells, it provides a unique tag to mark protein interaction sites. The adenine-methylated DNA fragments are next isolated using a specific endonuclease, and are sequenced after PCR amplification. This method allows the identification of LADs in diseases caused by mutations in A-type lamins (Guelen et al., 2008; Peric-Hupkes et al., 2010; Zullo et al., 2012; Perovanovic et al., 2016; Harr et al., 2020). In combination with the DamID technology, ^m6^A-Tracer method turns out to be a powerful tool to track LADs in living cells (Kind et al., 2013). ChIP-seq and DamID methods are both genomic DNA-binding profiling methods. However, DamID and ChIP-seq approaches each have their strengths and weaknesses. ChIP-seq method provides a snapshot of protein occupancy at a single location on DNA, whereas DamID method relies on DNA adenine methylation, which occurs over a period of several hours. Also, DamID method could give information of chromatin-binding events *in vivo*, whereas ChIP-seq method is only performed *in vitro* (Aughey et al., 2019). For a review of an elusive comparison between DamID and ChIP-seq approaches, please refer to (Aughey and Southall, 2016).

The arrangement of chromatin being intimately linked to the gene expression, it is of relevance to study the open state of the chromatin. This has been made possible by the assay for transposase-accessible chromatin with high-throughput sequencing (ATAC-seq) (Figure 4C **)**. This approach allows to map the chromatin accessibility at the genome-wide scale with an *in vitro* transposition of sequencing adaptors into native chromatin (Buenrostro et al., 2013; Grandi et al., 2022). Prokaryotic Tn5 transposase is integrated into accessible chromatin regions. The combination of sequencing adaptors and Tn5 transposase integration allows to simultaneously fragment and sequence the open chromatin regions (Buenrostro et al., 2015). ATAC-seq method can be used in combination with ChIP-seq and DamID to correlate LADs spatial organization and gene expression with a chromatin region opening (Lee et al., 2019). A recent methodology development, ATAC-see, allowed to visualize open chromatin regions, by replacing the sequencing by fluorescent adaptors (Chen et al., 2016).

It is also essential to visualize regions of the genome, in order to reveal their individual relationships with nuclear structures in single cells. This is achieved by DNA fluorescence *in situ* hybridization (FISH) and more recently, by 3D-FISH (FISH on 3D-preserved nuclei with immunofluorescence) in cells (Clements et al., 2016). 3D-FISH allows to study individual locus or discrete number of loci within the nucleus (Szabo et al., 2021). In 3D-FISH methods, (Figure 4D) DNA loci of interest are labeled with immunofluorescent DNA sequence specific or chromosome paint probes and visualized by fluorescent microscopy (Jensen, 2014). This approach is efficient to study the nuclear organization at the single-cell level. More recently, a quantitative high-resolution imaging approach, which combines FISH, array tomography imaging, and multiplexed immunostaining, has been implemented for investigating chromatin organization in complex tissues (Linhoff et al., 2015). 3D-FISH is thus a method of choice to study and visualize LADs and its location in *Cardiolaminopathy* (Kind et al., 2013; Paulsen et al., 2017; Bertero et al., 2019b; Salvarani et al., 2019).

## Chromatin reorganization in cardiolaminopathy

Specific cellular and molecular mechanisms of pathogenesis and progression of *Cardiolaminopathy* remain unclear and are still under investigation. Several hypotheses have been proposed attempting to link the pathophysiology of this disease to known or emerging functions of A-type lamins, among which the “chromatin hypothesis”. This model is based on the interaction between A-type lamins and chromatin via LADs. This hypothesis suggests that a cell-type specific chromatin reorganization caused by a disruption of A-type lamins protein expression in *Cardiolaminopathy*, leads to an abnormal gene expression and thus participates to the pathogenesis.

The “chromatin model” is supported by different studies. It emerges from recent works suggesting that *CardioLaminopathy* results from dysregulated gene expression as a consequence of LADs reorganization in cardiomyocytes. In fact, these LADs play a role for chromatin organization and gene expression regulation (Guelen et al., 2008; Shimi et al., 2008; Zullo et al., 2012; Solovei et al., 2013). It has been shown that the organization of the LADs is altered in *CardioLaminopathy* (Perovanovic et al., 2016; Shah et al., 2021)*.* Due to the interaction between A-type lamins and chromatin, dysregulated gene expression has emerged as a plausible mechanism to partially explain the pathogenesis of *CardioLaminopathy*. One study focused on the organization of LADs in *CardioLaminopathy* using ChIP-Seq approach and RNA-sequencing in explanted hearts from patients (Cheedipudi et al., 2019). The authors highlighted the role of LADs in the regulation of gene expression, and identified several transcription factors involved in biological processes, such as cell death/survival, cell cycle and metabolism (Cheedipudi et al., 2019)*.* These findings demonstrated that a reorganization of LADs associated with alteration of gene profile expression are occurring in *CardioLaminopathy*. This was recently strongly supported by a study from another group (Shah et al., 2021). This study was based on the differentiation of cardiomyocytes, adipocytes and hepatocytes derived from iPSCs from patients with *cardiolaminopathy*. The authors showed that mutations in A-type lamins result in abnormal gene regulation from peripheral chromatin regions only in cardiomyocytes cell types. With this study, Shah and collaborators provided evidence not only of the LADs reorganization in *Cardiolaminopathy* but also of the cell-type specific organization of several LADs and the link between LADs-chromatin interaction with cell identity. This study thus supports the tissue-specificity of the phenotypes observed in *Cardiolaminopathy*. Bertero and others studied chromosome conformation in cardiomyocytes derived from iPSCs from patient with *CardioLaminopathy* carrying the *LMNA* p. R225X mutation (Bertero et al., 2019b). The authors reported a switch from A- and B-compartments restricted at only ∼1% of the genome, resulting in transcriptional activation of genes shifting from nuclear periphery to the nuclear interior. The limited chromatin compartment change thus challenges the “chromatin model”, as chromosomal compartmentation may not be the primary pathogenic mechanism in *CardioLaminopathy*. The distribution of open chromatin was biased towards the nuclear periphery in cardiomyocytes derived from iPSCs from patient with *CardioLaminopathy* carrying the *LMNA* p. K117fs mutation, compared with isogenic control cells (Lee et al., 2019). Using a combination of ChIP-seq and ATAC-seq approaches (see section ‘methods to identify and visualize LADs’), Lee and collaborators have shown that the abnormal conformation of open chromatin is accompanied by an increase of open chromatin marks of the LADs gene promoters and a decrease of LADs association with A-type lamins. These results suggest that *LMNA* p. K117f mutation leads to local changes in LADs leading to transcriptional activation. Another study using cardiomyocytes derived from iPSCs from patient with *CardioLaminopathy* carrying the *LMNA* p. K219T mutation showed that conduction abnormalities are caused by repression of *SCN5A*, a gene encoding a sodium channel, due to epigenetics changes (Salvarani et al., 2019). *SCN5A* may be included in a LAD that shifts from the nuclear interior towards the nuclear periphery in *CardioLaminopathy* (Lund et al., 2013). Hence, epigenetic state of tissue-specific genes could lead to transcriptional silencing in LADs (Leemans et al., 2019). These data showed that even a slightly change in chromatin organization in mutant cells can still participates to phenotype observed in *Cardiolaminopathy*.

A-type lamins are mostly located at the nuclear periphery and bind LADs but a pool of these proteins can be associated with the open chromatin (euchromatin) (Gesson et al., 2014). Recently, two studies have shown an alteration of A-type lamins interaction with euchromatin in dilated cardiomyopathy (Zhang et al., 2021; Feng et al., 2022). Collectively, all these studies participate to understand the underlying mechanisms of the disease pathogenesis.

The mammalian genome is organized in different three-dimensional levels, which are finely regulated during normal and pathological development (Zheng and Xie, 2019). In this review, we focused on the LADs organization and reorganization in cardiac disease context but other levels of three-dimensional genome organization can be disrupted in cardiac diseases (Meaburn et al., 2007; Mewborn et al., 2010; Rosa-Garrido et al., 2017; Chapski et al., 2021). A better knowledge of the regulation at different three-dimensional scales of genome organization could then help to better understand the development of cardiac diseases.

This review targets the genome organization specifically in cardiomyocytes. However, studying other cardiac cell type present in the heart environment (*e.g.*, cardiac fibroblasts and endothelial cells) could participate to further understand the pathogenesis of *Cardiolaminopathy* (Mewborn et al., 2010; Perovanovic et al., 2016; Ramirez-Martinez et al., 2021).

## Open questions and future directions

Recent advances to study chromatin organization shed exciting new light on the pathogenesis of *CardioLaminopathy*. Here, we have outlined recent findings and methods that uncovered the role of chromatin compartment dysregulation in both mice and humans, giving novel insights into the nature of cardiomyocyte dysfunction during disease progression. Nevertheless, there is still a long list of open questions that needs to be answered. While recent studies have elegantly identified the role of chromatin regulation during the pathogenesis of *CardioLaminopathy*, the distinct roles of chromosomal compartmentation remain elusive. Application of selective tools targeting only the context-dependent A-type lamins gene expression by Cre recombinase-mediated gene targeting or defined drugs might provide new insights into the nature of chromatin organization in the heart. To date, little is known about the temporal regulation of chromatin organization and the transcriptional level during development of *CardioLaminopathy*. Analyzing chromatin organization and gene regulation in heart along the progression of the disease will illuminate another aspect of genome regulation and help to identify specialized functions for the pathogenesis of age-related disease. In addition, whether such chromatin phenotypes are detrimental or beneficial for disease progression still remain unclear. Answering such critical questions, together with profiling heterogeneous subclusters of chromatin organization in healthy and diseased situations, can open new avenues for the development of therapeutic targeting of cardiomyocytes in *CardioLaminopathy*. These studies open up new perspectives in an attempt to explain the pathophysiology of *Cardiolaminopathies* and pinpoint possible new A-type lamins functions still unexplored.
